# Ultrasound-detected bone erosion is a relapse risk factor after discontinuation of biologic disease-modifying antirheumatic drugs in patients with rheumatoid arthritis whose ultrasound power Doppler synovitis activity and clinical disease activity are well controlled

**DOI:** 10.1186/s13075-017-1320-2

**Published:** 2017-05-25

**Authors:** Shin-ya Kawashiri, Keita Fujikawa, Ayako Nishino, Akitomo Okada, Toshiyuki Aramaki, Toshimasa Shimizu, Masataka Umeda, Shoichi Fukui, Takahisa Suzuki, Tomohiro Koga, Naoki Iwamoto, Kunihiro Ichinose, Mami Tamai, Akinari Mizokami, Hideki Nakamura, Tomoki Origuchi, Yukitaka Ueki, Kiyoshi Aoyagi, Takahiro Maeda, Atsushi Kawakami

**Affiliations:** 1 Department of Community Medicine, Nagasaki University Graduate School of Biomedical Sciences, 1-12-4 Sakamoto, Nagasaki, 852-8523 Japan; 2 Department of Public Health, Nagasaki University Graduate School of Biomedical Sciences, Nagasaki, Japan; 3 Department of Immunology and Rheumatology, Nagasaki University Graduate School of Biomedical Sciences, Nagasaki, Japan; 4 Department of Internal Medicine, Isahaya General Hospital, Isahaya, Japan; 5 Department of Center for Comprehensive Community Care Education, Nagasaki University Graduate School of Biomedical Sciences, Nagasaki, Japan; 6 Department of Internal Medicine, Japanese Red Cross Nagasaki Genbaku Hospital, Nagasaki, Japan; 7 Center for Rheumatic disease, Sasebo Chuo Hospital, Sasebo, Japan

**Keywords:** Rheumatoid arthritis, RA, Ultrasound, Biologic disease-modifying antirheumatic drug, bDMARD, Remission, Discontinuation

## Abstract

**Background:**

In the present study, we explored the risk factors for relapse after discontinuation of biologic disease-modifying antirheumatic drug (bDMARD) therapy in patients with rheumatoid arthritis (RA) whose ultrasound power Doppler (PD) synovitis activity and clinical disease activity were well controlled.

**Methods:**

In this observational study in clinical practice, the inclusion criteria were based on ultrasound disease activity and clinical disease activity, set as low or remission (Disease Activity Score in 28 joints based on erythrocyte sedimentation rate <3.2). Ultrasound was performed in 22 joints of bilateral hands at discontinuation for evaluating synovitis severity and presence of bone erosion. Patients with a maximum PD score ≤1 in each joint were enrolled. Forty patients with RA were consecutively recruited (November 2010–March 2015) and discontinued bDMARD therapy. Variables at the initiation and discontinuation of bDMARD therapy that were predictive of relapse during the 12 months after discontinuation were assessed.

**Results:**

The median patient age was 54.5 years, and the median disease duration was 3.5 years. Nineteen (47.5%) patients relapsed during the 12 months after the discontinuation of bDMARD therapy. Logistic regression analysis revealed that only the presence of bone erosion detected by ultrasound at discontinuation was predictive of relapse (OR 8.35, 95% CI 1.78–53.2, *p* = 0.006). No clinical characteristics or serologic biomarkers were significantly different between the relapse and nonrelapse patients. The ultrasound synovitis scores did not differ significantly between the groups.

**Conclusions:**

Our findings are the first evidence that ultrasound bone erosion may be a relapse risk factor after the discontinuation of bDMARD therapy in patients with RA whose PD synovitis activity and clinical disease activity are well controlled.

## Background

It is now widely accepted that an ultrasound examination is superior to a clinical examination for the detection of joint inflammation [1–3]. The European League Against Rheumatism (EULAR) task force has published its recommendations for the use of imaging of the joints in the clinical management of rheumatoid arthritis (RA), and the recommendations state that ultrasound is very helpful for identifying synovitis and bone erosions and thus for making accurate diagnoses, predicting outcomes and responses to treatment, and monitoring disease progression [4]. In addition, ultrasound can be used to assess the persistent inflammation that predicts subsequent joint damage in patients achieving clinical remission [4].

Highly sensitive modalities such as ultrasound and magnetic resonance imaging (MRI) have revealed the presence of residual or subclinical synovitis in patients with RA in clinical remission [3, 5, 6]. A meta-analysis of reports of residual synovitis detected by ultrasound revealed that the prevalence of synovial hypertrophy with a power Doppler (PD) signal was 32% to 44% [6]. In addition, PD signal positivity of residual synovitis strongly predicted the risk of structural progression and relapse in clinical remission [3, 6].

The use of aggressive treatment, including biologic disease-modifying antirheumatic drugs (bDMARDs), has greatly improved outcomes for patients with RA. Although some patients showing persistent clinical remission may achieve bDMARD- and drug-free remission, a considerable proportion of the patients cannot maintain the clinical benefit [7–11]. There are no definitive predictive markers that enable the identification of such patients, and additional challenges have been to determine (1) how remission is maintained over time, (2) how subclinical disease can be detected and evaluated, and (3) how undetectable active or progressive disease can be distinguished from true remission of inflammation [12].

Another issue is the definition of imaging remission. Apart from mere clinical remission in terms of joint manifestations, imaging remission may have to be considered in RA cases in which synovial inflammation has also completely ceased [4, 12]. The lower relapse rates observed in patients with normal ultrasound results support the biological relevance of this concept [12]. Recent studies indicated that the presence of synovitis with a PD signal before the discontinuation or dose reduction of a bDMARD is an independent predictor for a subsequent relapse in patients in clinical remission [13, 14]. Therefore, if a discontinuation or dose reduction of a patient’s treatment with a bDMARD is being considered, it may be preferable to confirm that PD activity is absent or low by an ultrasound examination. However, relapses occur even in patients with RA without PD activity. These data suggest that the identification of ultrasound indices other than synovitis, especially PD synovitis, is necessary to improve the accuracy of ultrasound imaging remission. The objectives of this study were to explore potential risk factors of relapse, especially ultrasound indices, after the discontinuation of bDMARD therapy in patients with RA whose ultrasound PD synovitis activity and clinical disease activity were well controlled.

## Methods

### Patients

Forty patients with RA who fulfilled the 2010 RA classification criteria [15] and discontinued bDMARD therapy during the period from November 2010 to March 2015 were consecutively recruited to participate this study from three rheumatology centers in Nagasaki Prefecture (Nagasaki University Hospital, Isahaya General Hospital, and Japanese Red Cross Nagasaki Genbaku Hospital). All of the patients satisfied the following inclusion criteria. All patients (1) had been treated with a bDMARD for at least 6 months, (2) were in sustained low disease activity (LDA) or remission (i.e., with a Disease Activity Score in 28 joints based on erythrocyte sedimentation rate [DAS28-ESR] <3.2) for at least 3 months, and (3) and had not taken oral glucocorticoids at the discontinuation of bDMARD therapy. Each patient had undergone an ultrasound assessment of 22 joints of bilateral hands at discontinuation, and the maximum grade of PD at each joint was ≤1. All patients underwent clinical and laboratory assessments every month after discontinuation for 12 months. This study was a retrospective observational investigation using anonymized information. The participants had discontinued bDMARD therapy depending on the clinical decision before we approached to take part in this study. The patients gave their informed consent to be subjected to the protocol, which was approved by the institutional review board (reference number 10062546) of Nagasaki University Hospital.

### Clinical and laboratory assessments

Clinical and laboratory assessments included 28-joint swollen and tender joint counts; the evaluator global assessment (EGA); the patient global assessment (PtGA); the Health Assessment Questionnaire-Disability Index; the ESR; and the levels of C-reactive protein (CRP), matrix metalloproteinase 3 (MMP-3), rheumatoid factor (RF), and anticyclic citrullinated peptide antibody (ACPA). Clinical disease activity was evaluated by the DAS28-ESR, the Simplified Disease Activity Index (SDAI), and the Clinical Disease Activity Index (CDAI).

### Ultrasound assessment

The ultrasound examinations were performed by Japan College of Rheumatology (JCR)-certified sonographers (SK, KF, AN, TA, and TS). SK is also involved in the JCR Committee for the Standardization of Musculoskeletal Ultrasonography. A systematic multiplanar grayscale (GS) and PD examination of joints was performed with different scanners (Aplio 500, Toshiba, Tokyo, Japan; Ascendus, Hitachi, Tokyo, Japan; Noblus, Hitachi; or LOGIQ E9, GE Healthcare, Wauwatosa, WI, USA), using a multifrequency linear transducer (12–14 MHz). All of the scanners are high-end models. On each scanner, the factory setting for superficial musculoskeletal assessment was used. The settings were also adjusted for increasing Doppler sensitivity by decreasing the pulse repetition frequency (800 Hz) or velocity range (3.1 cm/second) and adjusting the Doppler gain to a level just below random noise. Thus, appropriate scanning conditions have been considered to be achieved even if different scanners areobtained. Articular synovitis was assessed by ultrasound at the 22 joints, including the bilateral wrist joints (radiocarpal, intercarpal, and distal radioulnar joints) as well as the first to fifth metacarpophalangeal (MCP) and proximal interphalangeal (PIP) joints. Each joint was scored for GS as well as PD on a scale from 0 to 3 [16], and the sum of the GS or PD scores was used as the indicator of ultrasound disease activity. In previous studies, we demonstrated that the present synovitis scoring system was useful for evaluating the early diagnosis and therapeutic effectiveness of medications for patients with RA [5, 17–19]. Positivity of GS and PD in articular synovitis was defined as a score ≥1 for GS and PD, respectively.

We also examined the presence or absence of bone erosion in the present study. In addition to synovitis, ultrasound bone erosion appears to be predictive of further radiographic progression [4]. Erosion was defined by a cortical break seen in two perpendicular planes [20]. Clearly visible erosions in both longitudinal and transverse scans were considered for the study. We scanned the dorsal, volar, and lateral aspects of the involved joints (wrist, MCP, or PIP). The sonographer paid particular attention in assessing unifocal bony breaks of small size to avoid misinterpretation with anatomical necks or vascular bone channels [21, 22]. Vascular bone channels were distinguished from bone erosion on the basis of anatomical location, insertion of feeding vessels detected by PD, and absence of adjacent synovial lesion. All joint regions were sonographically examined in a standardized manner according to the EULAR [23] and JCR guidelines on a semiquantitative scale as described previously [16].

### Definition of relapse

Patients were assessed monthly by JCR-certified rheumatologists and received routine clinical management after the discontinuation of bDMARD therapy as described above. Patients were considered to have had a relapse when their antirheumatic treatment was escalated because the physician concluded that the patient was exhibiting an increase in RA disease activity. Although the physicians had recognized the ultrasound findings at discontinuation of bDMARD therapy, they did not decide on whether a relapse had occurred solely on the basis of ultrasound findings.

### Statistical analyses

Within-group comparisons were made using the Mann-Whitney *U* test or the χ^2^ test. We performed a logistic regression analysis to investigate the relationships between the variables at the initiation or discontinuation of the bDMARDs and relapse during the 12 months after the discontinuation of the patient’s bDMARD treatment. For the multivariate logistic regression analysis, we selected the variables that showed *p* values <0.2 (Table 1). The overall significance level for the statistical analysis was 5% (two-sided). *p* Values <0.05 were considered significant.

**Table 1:** Comparison of clinical characteristics and ultrasound findings between relapse and nonrelapse patients

	Relapse (*n* = 19)	Nonrelapse (*n* = 21)	*p* Value
Clinical characteristics at initiation of bDMARD			
Positivity of RF, *n*	16 (84.2)	18 (85.7)	0.89
Positivity of ACPA, *n*	18 (94.7)	19 (90.5)	0.61
DAS28-ESR	4.76 (3.99–5.15)	4.02 (3.60–5.09)	0.26
SDAI	19.6 (15.2–26.1)	14.7 (11.1–24.6)	0.19
CDAI	18 (13.5–24.5)	14.4 (11.0–20.8)	0.28
Clinical characteristics at discontinuation of bDMARD			
Age, years	55 (46–61)	53 (41–61)	0.95
Female sex	14 (73.7)	19 (86.4)	0.16
Duration of disease, years	5.0 (1.4–8.0)	3.0 (2.0–4.0)	0.54
Positivity of RF, *n*	11 (64.7)	12 (57.1)	0.74
bDMARD use, *n*	IFX, 8; ADA, 5; CZP, 1; ETN, 2; TCZ, 2; ABT, 1	IFX, 6; ADA, 2; GLM, 4; CZP, 1; ETN, 1; TCZ, 5; ABT, 2	
TNF inhibitor use, *n*	16 (84.2)	14 (66.7)	0.20
Concomitant MTX, *n*	17 (89.5)	16 (76.2)	0.27
Duration of bDMARD therapy, months	12 (10–23)	12 (10–21)	0.75
Duration until clinical remission, months	3 (1–5)	3 (2–4)	0.95
Duration of clinical remission, months	8 (7–16)	9 (6–12)	0.80
Tender joint counts, *n*/28 joints	0 (0)	0 (0)	0.33
Swollen joint counts, *n*/28 joints	0 (0)	0 (0)	0.61
PtGA, mm	3 (2–7)	4 (2–8)	0.62
EGA, mm	3 (2–4)	4 (2–5)	0.39
CRP, mg/dl	0.05 (0.03–0.05)	0.05 (0.05–0.09)	0.41
ESR, mm/h	11 (8–15)	7 (5–14)	0.44
MMP-3, ng/ml	35 (26–58)	33 (28–49)	0.77
DAS28-ESR	1.73 (1.29–2.01)	1.57 (1.20–2.02)	0.76
SDAI	0.9 (0.5–1.2)	0.8 (0.5–3.0)	0.58
CDAI	0.8 (0.5–1.1)	0.7 (0.4–2.7)	0.62
HAQ-DI	0 (0–0.1)	0 (0)	0.67
Ultrasound findings			
Positivity of GS, *n*	10 (52.6)	14 (66.7)	0.37
Total GS score	2 (0–4)	2 (0–4)	0.65
Positivity of PD, *n*	5 (26.3)	3 (14.3)	0.34
Total PD score	0 (0)	0 (0)	0.45
Positivity of bone erosion, *n*	10 (52.6)	3 (14.3)	0.01

*Abbreviations: ACPA* Anticyclic citrullinated peptide antibody, *bDMARD* Biologic disease-modifying antirheumatic drug, *CDAI* Clinical Disease Activity Index, *CRP* C-reactive protein, *DAS28* Disease Activity Score in 28 joints, *EGA* Evaluator global assessment, *ESR* Erythrocyte sedimentation rate, *GS* Grayscale, *HAQ-DI* Health Assessment Questionnaire Disability Index, *MMP-3* Matrix metalloproteinase-3, *MTX* Methotrexate, *PD* Power Doppler, *PtGA* Patient global assessment, *RF* Rheumatoid factor, *SDAI* Simplified Disease Activity Index *TNF* Tumor necrosis factor, *IFX* Infliximab, *ADA* Adalimumab, *CZP* Certolizumab pegol, *TCZ* Tocilizumab, *ABT* Abatacept, *GLM* Golimumab

The data are median (interquartile range, Q_1-4_–Q_3/4_) or as number (percent)

## Results

### Patient characteristics at discontinuation of bDMARD therapy

Forty patients (33 females, 7 males) were enrolled in this study. The median (IQR [Q_1-4_-Q_3/4_]) patient age was 54.5 (45.8–61.3) years, and the median disease duration was 3.5 (5.5) years. The median (IQR) values were as follows: tender joints count 0 (0), swollen joint count 0 (0), PtGA 4 (2–8) mm, EGA 3 (2–5) mm, ESR 10 (5–15) mm/h, CRP 0.05 (0.03–0.06) mg/dl, MMP-3 33 (26–55) ng/ml, DAS28-ESR 1.63 (1.25–2.01), SDAI 0.9 (0.5–1.4), and CDAI 0.8 (0.4–1.3). The rate of remission (DAS28-ESR less than −2.6) was 92.5%. The rate of absence of PD (total PD score 0) was 80%. Bone erosion was detected in 24 joints (17 wrist and 7 MCP joints) and in 13 patients. It was not detected by conventional radiography in 10 (41.7%; 5 wrist and 5 MCP joints) of 24 joints and 6 (46.2%) of 13 patients. Thirty patients had been treated with tumor necrosis factor (TNF) inhibitors, and the other ten patients had been treated with non-TNF inhibitors.

### Relapse during the 12 months after discontinuation of bDMARD therapy

**Fig. 1:**
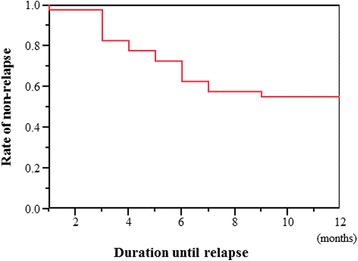
Kaplan-Meier survival estimate

Nineteen (47.5%) patients relapsed during the 12 months after discontinuation of their bDMARD therapy. The time course of the relapse-free periods by Kaplan-Meier survival estimate is shown in Fig. 1. Among the patients who relapsed, most (15 of 19 patients [78.9%]) relapsed within 6 months after bDMARD discontinuation. Treatment with a bDMARD was reintroduced in 13 patients. Treatment with conventional disease-modifying antirheumatic drugs (cDMARDs) was increased for three patients and added for two patients. A low-dose corticosteroid was added for one patient.

### Comparison of clinical characteristics and serologic biomarkers at initiation and discontinuation of bDMARD therapy between relapse and nonrelapse patients

As shown in Table 1, no clinical variables, including seropositivity, composite measurements, and treatments at initiation and discontinuation, were significantly different between the relapse patients and nonrelapse patients.

### Comparison of ultrasound findings at discontinuation of bDMARD therapy between relapse and nonrelapse patients

The positivity and the total scores of both GS and PD were not significantly different between the relapse patients and the nonrelapse patients. However, the positivity of bone erosion detected by ultrasound was significantly higher in the relapse patients than in the nonrelapse patients (52.6 vs. 14.3%, *p* = 0.01).

### Multivariate regression analysis

We performed multivariate logistic regression analysis to identify any variables that could be used to predict relapse during the 12 months after discontinuation of bDMARD therapy (Table 2). Only the presence of bone erosion detected by ultrasound at bDMARD discontinuation was an independent predictive variable for relapse (OR 8.35, 95% CI 1.78–53.2, *p* = 0.006).

**Table 2:** Multivariate regression analysis to predict relapse during the 12 months after discontinuation of biologic disease-modifying antirheumatic drug therapy

	Comparison	OR	95% CI		*p* Value
Sex	Female vs. male	0.25	0.02	1.79	0.17
SDAI at initiation of bDMARD	1 increase	0.95	0.88	1.03	0.20
Bone erosion by ultrasound	Presence vs. absence	8.35	1.78	53.2	0.017

*bDMARD* Biologic disease-modifying antirheumatic drug; *SDAI* Simplified Disease Activity Index

Variables with a *p* value less than 0.02 in Table 1 were used in multivariate models

## Discussion

Our study results show that the presence of bone erosion detected by ultrasound at bDMARD discontinuation was the only independent risk factor of relapse during the 12 months after discontinuation of bDMARD therapy. No other factors, including clinical variables at initiation, clinical and ultrasound variables at discontinuation, and biomarkers, were associated with relapse.

Several randomized control trials of bDMARD strategies addressing the discontinuation or dose reduction of bDMARDs have been reported [7, 8]. The PRESERVE trial, as a representative study, was undertaken to determine whether LDA could be sustained with discontinuation or dose reduction of etanercept (ETN) in patients with established RA [9]. After treatment with 50 mg of ETN plus methotrexate (MTX) for 36 weeks, patients were randomized to three groups: 50 mg of ETN plus MTX (maintenance), 25 mg of ETN plus MTX (reduction), and placebo plus MTX (discontinuation). At 12 months after randomization, LDA had been maintained in only 42.6% of the discontinuation group but in 82.6% of the maintenance group and 79.1% of the reduction group [9]. Similarly, on the basis of results of registries in the United States [10] and Japan [11], clinical benefit could not be maintained in a considerable portion of the patients after discontinuation of bDMARD therapy.

Regarding the significance of subclinical synovitis in patients with RA [3, 5, 6], we reported that moderate or severe PD activity was present in approximately 30% of patients who were free of physical synovitis [5]. In these physical synovitis-free patients, the use of bDMARDs was low and the presence of ultrasound-detected bone erosion was high in the group with moderate or severe PD activity [5]. A strategy combining clinical and ultrasound assessments may better select individuals for sustained discontinuation or dose reduction of DMARD therapy. In this regard, three prospective studies that included ultrasound indices reported predictive factors of relapse after discontinuation or dose reduction of bDMARD therapy in sustained clinical remission [13, 14, 24]. Among these factors, higher clinical disease activity and a higher PD synovitis score are thought to be important indicators of the failure of bDMARD discontinuation or dose reduction.

The difference between these studies and our present data is that neither clinical disease activity nor ultrasound synovitis activity was associated with discontinuation of bDMARDs. Compared with these studies, the clinical disease activity and the residual ultrasound synovitis activity at entry in the present study were quite low, even in the patients who experienced subsequent relapse [13, 14, 24]. Therefore, these factors would not predict relapse in the present patients. However, although no statistical significance was found between the relapse patients and nonrelapse patients, the frequency of PD grade 1 synovitis was numerically high in the relapse patients compared with nonrelapse patients. Thus, no PD signal, instead of PD grade at each joint being ≤1, might be crucial to maintaining clinical remission after discontinuation of bDMARD therapy. As stated in the discussion of limitations below, a study including a larger number of patients is necessary to confirm this speculation.

The novel aspect of the present study was the evaluation of bone erosion by ultrasound in patients with RA whose ultrasound PD synovitis activity was well controlled. The presence of ultrasound bone erosion at discontinuation was the only independent predictor of relapse during the 12 months after bDMARD therapy. We speculate that ultrasound-detected bone erosion is important for the prediction of relapse for the reasons outlined below.

First, we and other investigators have found a close association between the presence of bone erosion and residual PD synovitis at the joint level in patients with RA in clinical remission or with LDA [5, 25]. Thus, bone erosions are the natural consequence of persistent joint inflammation. In this regard, MRI-detected bone edema (osteitis), which is bone inflammation that is strongly predictive of further radiographic progression [26, 27], coexisted with PD synovitis in patients with RA [28]. We suspect that ultrasound can detect subclinical findings of joint inflammation in the areas most aggressively hit by the disease and/or where the inflammation has started earlier where bone erosion is detected. Alternatively, ultrasound-detected bone erosion could be derived from the integration of joint inflammation, whereas PD synovitis might always not be detected at bDMARD discontinuation.

Second, the presence of bone degradation elements as a result of joint damage can trigger immunological pathways, developing further joint inflammation, because it is well known that bone erosion detected by ultrasound or plain radiography can be used for the prediction of further joint destruction [25, 29]. These two hypotheses do not conflict, and we thus speculate that there is a vicious cycle in which the joint inflammation leads to joint damage, with the consequent release of bone and cartilage fragments that sustain the joint inflammation, resulting in further relapse.

With respect to serum biomarkers, the best-studied predictor of relapse to date is ACPA positivity [12]. In the REduction of Therapy in RA patients in Ongoing remission (RETRO) study, ACPA status clearly indicated a higher relapse risk with lower chances of maintaining remission when ACPAs are present [12]. Data derived from other investigations, such as the Dutch Behandel Strategieen (BeSt) study and the High Induction Therapy with Anti-Rheumatic Drugs (HIT HARD) study, support this concept [12]. In contrast, we did not find an association of positivity for ACPA or RF at the initiation or the discontinuation of bDMARDs with subsequent relapse. In the Remission Induction by Remicade in RA (RRR) study, the continuous presence of RF was reported to lower the likelihood of a successful withdrawal of TNF inhibitors [12]. The serial changes of positivity for ACPA or RF after the discontinuation of bDMARDs should thus be studied further. In addition, the titer of autoantibodies might be important to predict RA relapses.

Some limitations of this study should be acknowledged. The RA population was relatively small and heterogeneous regarding the patients’ RA characteristics, bDMARDs, and dosages. The limited sample size of our study does not allow for subanalyses of differences between bDMARDs with different modes of action. A study with a larger number of patients and a longer follow-up period is needed to establish optimal ultrasound-based strategies to reduce the unnecessarily long use of bDMARDs.

## Conclusions

The results of our study suggest that a risk of relapse after the discontinuation of bDMARD therapy is high in patients whose conditions are suggestive of already-formulated ultrasound-detected joint damage (i.e., bone erosion), even if their clinical and ultrasound synovitis activities are well controlled. Therefore, lower relapse rates may be attained with earlier administration of bDMARD therapy, before joint destruction appears.

## Abbreviations

ABT: Abatacept
ACPA: Anticyclic citrullinated peptide antibody
ADA: Adalimumab
bDMARD: Biologic disease-modifying antirheumatic drug
CDAI: Clinical Disease Activity Index
cDMARD: Conventional disease-modifying antirheumatic drug
CRP: C-reactive protein
CZP: Certolizumab pegol
DAS28: Disease Activity Score in 28 joints
EGA: Evaluator global assessment
ESR: Erythrocyte sedimentation rate
ETN: Etanercept
EULAR: European League Against Rheumatism
GLM: Golimumab
GS: Grayscale
HAQ-DI: Health Assessment Questionnaire Disability Index
IFX: Infliximab
JCR: Japan College of Rheumatology
LDA: Low disease activity
MCP: Metacarpophalangeal
MMP: Matrix metalloproteinase 3
MRI: Magnetic resonance imaging
MTX: Methotrexate
PD: Power Doppler
PIP: Proximal interphalangeal
PtGA: Patient global assessment
RA: Rheumatoid arthritis
RF: Rheumatoid factor
SDAI: Simplified Disease Activity Index
TCZ: Tocilizumab
TNF: Tumor necrosis factor
